# Responses of Leaky Integrate-and-Fire Neurons to a Plurality of Stimuli in Their Receptive Fields

**DOI:** 10.1186/s13408-016-0040-2

**Published:** 2016-05-23

**Authors:** Kang Li, Claus Bundesen, Susanne Ditlevsen

**Affiliations:** Department of Mathematical Sciences, University of Copenhagen, Universitetsparken 5, Copenhagen, 2100 Denmark; Department of Psychology, University of Copenhagen, Øster Farimagsgade 2A, Copenhagen, 1353 Denmark

**Keywords:** Probability-mixing, Response-averaging, Parameter estimation, Model selection, Visual attention

## Abstract

A fundamental question concerning the way the visual world is represented in our brain is how a cortical cell responds when its classical receptive field contains a plurality of stimuli. Two opposing models have been proposed. In the response-averaging model, the neuron responds with a weighted average of all individual stimuli. By contrast, in the probability-mixing model, the cell responds to a plurality of stimuli as if only one of the stimuli were present. Here we apply the probability-mixing and the response-averaging model to leaky integrate-and-fire neurons, to describe neuronal behavior based on observed spike trains. We first estimate the parameters of either model using numerical methods, and then test which model is most likely to have generated the observed data. Results show that the parameters can be successfully estimated and the two models are distinguishable using model selection.

## Introduction

The receptive field of a neuron in the visual system can be defined as the spatial area in which stimulation changes the firing pattern of the neuron. In primary visual cortex, receptive fields are small, with typical values of, for example, 0.5–2 deg of visual angle near the fovea. Moving up the hierarchy of extrastriate visual areas along either the dorsal [1] or the temporal [2] pathway, receptive field sizes grow substantially [3, 4], reaching, for example, a value of about 30 deg in the inferotemporal cortex. A plausible explanation is that since these areas process more complex aspects of the visual environment, information has to be integrated over larger spatial areas, such as when encoding faces [5] in the ventral pathway or optic flow patterns [6] in the dorsal one. Typically, receptive fields that are so big will contain a plurality of distinct stimulus objects rather than just a single stimulus object [7]. The way a cortical cell responds when its classical receptive field contains a plurality of stimuli is a basic question concerning the way the visual world is represented in our brain.

### Probability-Mixing and Response-Averaging

In a pioneering study, Reynolds et al. [8] found that a typical cell in visual area V2 or V4 in monkeys responded to a pair of objects in its classical receptive field by adopting a rate of firing which, averaged across trials, equaled a weighted average of the responses to the individual objects when these were presented one at a time, with greater weight on an object the more attention was directed to the object. Reynolds et al. accounted for their data by proposing that on each individual trial, the firing rate of a cell to a plurality of stimulus objects equaled a weighted average of the firing rates to the individual objects when these were presented alone. Bundesen et al. [9, 10] proposed an alternative explanation of the data of Reynolds et al. by pointing out that the effects observed in firing rates that were averaged across trials could be explained by assuming that on each individual trial, when a plurality of objects were presented, the cell responded as if just one of the objects was presented alone, so that across trials, the response of the cell was a probability mixture of the responses to the individual objects when these were presented alone.

In the *response-averaging* model proposed by Reynolds et al. [8] (see also [11–18]), the neuron responds with a weighted average of the responses to single stimuli. By contrast, in the *probability-mixing* model proposed by Bundesen et al. [9], the neuron responds at any given time to only one of the single stimuli with certain probabilities. Suppose the stimulus $S(t)$ presented to the neuron consists of *K* separated single stimuli, denoted by $S_{1}(t) , \ldots, S_{K}(t)$. In the response-averaging model, the neuron responds with a weighted average of responses to single stimuli, $\sum_{k} \beta_{k} I_{k}(t)$, with $\beta_{k}$ being the weights, and $\sum_{k}\beta_{k}=1$. Here $I_{k}(t)$ denotes the effects that $S_{k}$ has on the spiking neuron model, which we set to be the stimulus current. In the probability-mixing model, the response of the neuron equals one of the responses the neuron would have had if only a single stimulus was presented according to a probability mixture with probabilities $\alpha_{1}, \ldots, \alpha_{K}$, and $\sum_{k} \alpha_{k}=1$.

In our previous study [19], we compared the abilities of the probability-mixing model and the response-averaging model to account for spike trains (i.e., times of action potentials obtained from extracellular recordings) recorded from single cells in the middle temporal visual area (MT) of rhesus monkeys. Point processes were employed to model the spike trains. Results supported the probability-mixing model.

In this article, we combine the probability-mixing and the response-averaging model with the leaky integrate-and-fire (LIF) model, to describe neuronal behavior based on observed spike trains. This is cast in a general setting, where the stimulus $S(t)$ is represented as an input current to the neuron. The spike train data are simulated using the LIF model, responding either to a single stimulus or to a stimulus pair. In the case of stimulus pair, both response averaging and probability mixing are used. The first goal of the paper is to estimate parameters of either of the two models from spike train data. The second goal is to test which of the two models are most likely to have generated the observed data.

### The Leaky Integrate-and-Fire Model

The LIF models have been extensively applied to model the membrane potential evolution in single neurons in computational neuroscience (for reviews, see [20, 21]). The model has some biophysical realism while still maintaining mathematical simplicity. The simplest LIF model is an Ornstein–Uhlenbeck (OU) process with constant conductance, leak potential, and diffusion coefficient. More biophysical realism can be obtained by allowing for post-spike currents generated by past spikes [22]. Here we use post-spike currents generated via three types of kernels [23, 24]: bursting, decaying, and delaying kernel, all modeled by the difference between two decaying exponentials, but any kernel could be used.

### Temporal Stimulus

Constant stimuli are simple to handle and are widely used in both experiments and modeling work. However, real world stimuli are generally time varying. If they for example contain oscillatory components, the generated spike trains might also contain oscillations in the firing rates. Here we use three types of stimuli: oscillatory stimuli described by sinusoidal functions, pulsing stimuli modeled by piecewise constant functions, and stochastic stimuli described by OU processes.

### Method Summary

We combine the models describing neuronal response to a plurality of stimuli, namely the probability-mixing model and the response-averaging model, with the LIF framework, for different types of stimuli and response kernels. Parameter estimation is done by maximum likelihood using first-passage time probabilities of diffusion processes [25]. We solve the first-passage time problem by numerically solving either a partial differential equation (PDE), the Fokker–Planck equation, or an integral equation (IE), the Volterra integral equation. Numerical solutions of these equations have been extensively explored and applied in the computations of neuronal spike trains [26–28]. Inspired by these previous studies, we apply four numerical methods, including two Fokker–Planck related PDEs and two kinds of Volterra IEs, and compare the performance of the four methods. We also describe and compare two alternative methods for maximizing the likelihood function of the probability-mixing model, which are direct maximization of the marginal likelihood and the expectation–maximization (EM) algorithm. Finally, we show that the probability-mixing model and the response-averaging model can be distinguished in the LIF framework, by comparing parameter estimates and through uniform residual tests.

## Leaky Integrate-and-Fire Model with Stimuli Mixtures

The evolution of the membrane potential is described by the solution to the following stochastic differential equation:

1 $$ \begin{aligned} dX(t) &= b\bigl(X(t),t\bigr)\, dt + \sigma \, dW(t) \\ \hphantom{dX(t)}&= \bigl(-\gamma\bigl(X(t) - \mu\bigr) + I(t) + H(t) \bigr)\, dt + \sigma\, dW(t), \\ X(0) &= x_{0} ;\qquad X\bigl(t_{j}^{+}\bigr)= x_{0}, \\ t_{j} &= \inf\bigl\{ t > t_{j-1} : X(t) = x_{\mathrm{th}} \bigr\} \quad \mbox{for } j \geq1, t_{0}=0, \end{aligned} $$

where $t_{j}^{+}$ denotes the right limit taken at $t_{j}$. The drift term $b(\cdot)$ contains three currents: the leak current $-\gamma(X(t)-\mu)$, where *γ* is the decay rate and *μ* is the reversal potential, the stimulus-driven current $I(t)$, and the post-spike current $H(t)$. The potential $X(t)$ evolves until it reaches the threshold, $x_{\mathrm{th}}$, where it resets to $x_{0}$. Since the membrane potential $X(t)$ is not observed, but only the spike times $d=(t_{1}, t_{2}, \ldots)$, we can use any values for threshold and reset suitable for the numerical calculation. The noise is described by the standard Wiener process, $W(t)$, and the diffusion parameter, *σ*. The interspike intervals (ISIs) are defined by $t_{j+1}-t_{j}$.

The stimulus current $I(t)$ is shaped from the external stimulus current through a stimulus kernel $k_{s}(t)$ as $I(t)=\int_{-\infty}^{t}k_{s}(t-s)S(s)\, ds$, where $S(s)$ denotes the external current at time *s*. Similarly, the post-spike current arises from past spikes through a response kernel $k_{h}(t)$ by $H(t)=\int_{-\infty}^{t}k_{h}(t-s)\mathbb{I}(s)\, ds$. Here $\mathbb{I}(s)=\sum_{\tau\in d}\delta(s-\tau)$ describes the spike train, where $\delta(\cdot)$ denotes the Dirac delta function.

In this work, the stimulus kernel is assumed without memory, such that $k_{s}(t) = \delta(t)$. Then the stimulus current $I(t)$ is completely determined by the stimulus at time *t*, e.g., $I(t)=S(t)$. The response kernel is assumed to be the difference of two exponentials decaying over time,

2 $$ k_{h}(t)=\eta_{1}e^{-\eta_{2}t}- \eta_{3}e^{-\eta_{4}t} $$

with four positive parameters, $\eta= (\eta_{1},\eta_{2},\eta_{3},\eta_{4})$. By adjusting the parameters, different kernels are obtained. Note that in practice the four parameters are not identifiable, because different parameter sets can result in very similar kernels. Therefore, when we later verify parameter estimates we will not check each individual estimate, but only plot the estimated shape of the kernel function, which is the quantity of interest.

**Fig. 1:**
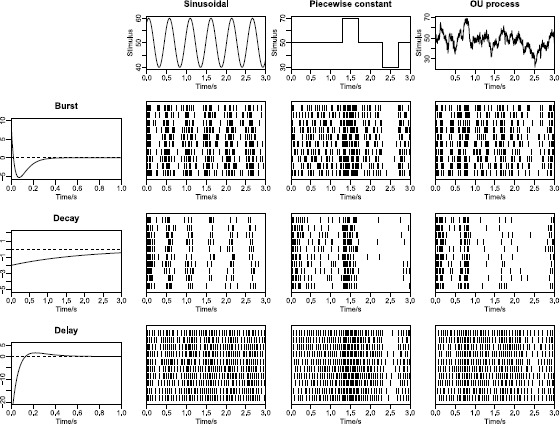
Realization of spike trains for different combinations of response kernels and stimuli. *Top panels* show the three stimulus types; sinusoidal, piecewise constant and Ornstein–Uhlenbeck process. *Left panels* show the burst, decay and delay response kernels. The *nine middle panels* illustrate spike train patterns for the different combinations of response kernels and stimuli. The patterns produced by each response kernel are apparent; the bursts of spikes for the burst kernel, the firing rate adaptation of the decay kernel, and the refractory period by the delay kernel (no short ISIs). Likewise, the patterns produced by each stimulus are apparent; periodicity by the sinusoidal, abrupt changing intensities by the piecewise constant, and slowly fluctuating changes in intensity by the random stimulus

Three types of kernels are used, shown in the left panels of Fig. 1. The *bursting* kernel is characterized by being positive in the beginning, then turning negative, and finally converging toward 0, which happens when $\eta_{1}>\eta_{3}$ and $\eta_{2}>\eta_{4}$. It follows that the most recent spikes have excitatory effects for the current spike probability, but the accumulation of past spikes has inhibitory effects, resulting in rhythmic spiking with bursts. The *decaying* kernel only has one negative exponential by setting $\eta_{1} = 0$. The parameters $\eta_{3}$ and $\eta_{4}$ are small such that the inhibitory effects are small but long-lasting, making the firing rate decay slowly over time. The *delaying* kernel has parameters $\eta _{1} < \eta_{3}$ and $\eta_{2} < \eta_{4}$. It is negative in the beginning, then turns positive, and finally converges to 0. The most recent spikes have inhibitory effects, neutralized later on by the accumulation of excitatory effects, resulting in delaying the immediate formation of a new spike after a spike, preventing short ISIs, which models the refractory period. In the center panels example spike trains for the different kernels and different stimuli are illustrated.

### Current from Stimulus Mixture

Suppose that inside the receptive field of the neuron there are at least two separated non-overlapping stimuli, which we will call a stimulus mixture. According to the probability-mixing model [9], the neuron responds to only one stimulus at any given time with certain probabilities. Thus, for a total of *K* stimuli, the stimulus-driven current, $I(t)$, follows a probability mixture:

3 $$ I(t) = S_{k}(t), \quad \mbox{with probability } \alpha_{k} $$

for $k=1,\ldots,K$ and $\sum_{k=1}^{K}\alpha_{k}=1$. Recall that the stimulus kernel $k_{s}(t) = \delta(t)$ and thus, the current caused by the *k*th stimulus $I_{k}(t)=S_{k}(t)$. According to the response-averaging model [11], the current is a weighted average of all stimuli currents:

4 $$ I(t) = \sum_{k=1}^{K} \beta_{k}S_{k}(t). $$

The leak current and the spike response current do not depend on the stimuli.

In the top panels of Fig. 1 three types of stimuli are illustrated. A *sinusoidal* stimulus is defined by

5 $$ S(t)=s_{1}\sin(s_{2}t+s_{3}) + s_{4} $$

with four parameters $s_{\mathrm{sin}}=(s_{1},s_{2},s_{3},s_{4})$ describing the stimulus. Note that it also covers a constant stimulus for $s_{1}=0$. A *piecewise constant* stimulus is defined by

6 $$ S(t) = \left \{ \textstyle\begin{array}{l@{\quad}l} s_{1}, & t_{1} \le t < t_{2}, \\ s_{2}, & t_{2} \le t < t_{3}, \\ \ldots, \\ s_{n}, & t_{n} \le t < t_{n+1} , \end{array}\displaystyle \right . $$

with parameters $s_{pw} = (s_{1}, s_{2}, \ldots, s_{n}, t_{1}, t_{2}, \ldots, t_{n+1}) $. A *stochastic* stimulus is given by an OU process described by the SDE:

7 $$ dS(t) = \bigl(s_{1} - S(t)\bigr) \, dt + s_{2}\, dW(t) $$

with two parameters $s_{\mathrm{OU}} = (s_{1}, s_{2})$. We assume throughout that the stimuli currents are known. Spike patterns from combinations of different types of stimuli and response kernels are shown in Fig. 1. Clear bursting, decaying and delaying effects can be seen.

**Fig. 2:**
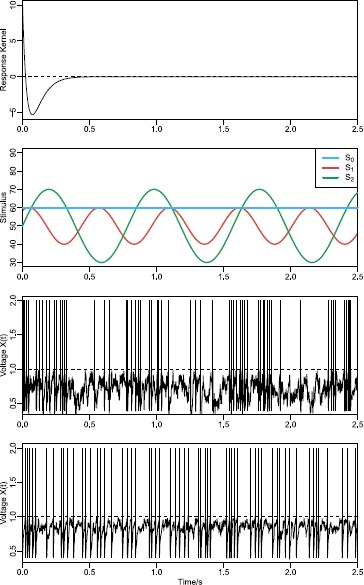
Illustration of voltage traces resulting from a bursting response kernel and sinusoidal stimuli. **(a)** Bursting response kernel in Eq. (2) with parameters $\eta =(50,25,40,15)$. **(b)** Examples of sinusoidal stimuli in Eq. (5). *Blue*: constant with $s_{0}=(0,\cdot,\cdot,60)$. *Red*: $s_{1}= (10, 12, 1, 50)$. *Green*: $s_{2}=(20, 8, 0, 50)$. **(c)** An example realization of membrane potential evolution, Eq. (1), responding to the sinusoidal signal $s_{1}$, and **(d)** responding to the constant signal $s_{0}$

Two example spiking patterns together with their voltage traces generated from either a sinusoidal or a constant stimulus together with a bursting post-spike kernel are shown in Fig. 2. There are bursts of spikes occasionally even under constant stimulus caused by the bursting response kernel. A sinusoidal stimulus causes long bursts, and in addition, the bursting kernel causes a clear separation of small burst periods also within the long bursting period.

## Maximum Likelihood Estimation Using First-Passage Time Probabilities

Our objective here is to estimate the parameters *μ* and *σ* from (1), the response kernel function $k_{h}$ in (2) represented by the parameter vector *η*, and either the probability vector of the stimuli in the mixture, $\alpha= (\alpha_{1}, \ldots, \alpha_{K})$, under the probability-mixing model, or the vector of weights in the average, $\beta= (\beta_{1}, \ldots, \beta_{K})$, in the response-averaging model. The estimation of the decay rate *γ* is difficult when there is no access to the membrane potential, but only spike times are observable, as discussed in [29, 30]. We therefore assume *γ* is known. The vector of all parameters in the model is thus *θ*, where $\theta=(\mu, \sigma, \eta, \alpha)$ in the probability-mixing model, and $\theta =(\mu, \sigma, \eta, \beta)$ in the response-averaging model. The stimulus is assumed known and the stimulus parameter vector *s* is therefore not estimated.

A similar LIF model with different stimulus and response kernels on single piecewise constant stimuli was used in Paninski et al. [24]. They showed that parameters can be estimated using MLE by solving the Fokker–Planck equation, covering also discussion of non-white noise and interneuronal interactions. The model was later applied to experimental data collected from retina of macaque monkeys [31]. Here we estimate parameters in the LIF model for various temporal stimuli and different response kernels, using four different numerical methods to calculate the likelihood function, within the framework of either the probability-mixing or the response-averaging model.

Suppose we observe *N* spike trains, $D=(d_{1}, \ldots, d_{N})$, all responding to the same stimulus mixture, where the *i*th spike train consists of $N_{i}$ spike times, $d_{i}=(t_{1}^{i}, \ldots, t_{N_{i}}^{i})$. The *j*th ISI of the *i*th spike train is then given by $t^{i}_{j+1}-t^{i}_{j}$. Assume that each measured spike train, i.e., each trial, is sufficiently short, such that, under the probability-mixing model, the neuron is only responding to one stimulus within the stimulus mixture, not switching the response within the trial.

### First-Passage Times and Probability Distributions

Modeling the spike train data as threshold crossings of the underlying diffusion process representing the unobserved membrane potential belongs to the so-called first-passage time problem [32, 33]. For models with no effects from past spikes, such that ISIs are assumed i.i.d., one approach is to build loss functions using the Fortet equation [29, 30]; see also [34]. A more general method, which allows for the post-spike effects in model (1), is to use maximum likelihood estimation (MLE) from numerical solutions of PDEs or IEs for the conditional distribution of the spike times or equivalently, the ISIs.

We use the following notation for the probability density functions (PDFs) and cumulative distribution functions (CDFs) of interest:

$$\begin{aligned} f\bigl(x, t | \mathcal{H}_{t},\theta,S(t)\bigr) &\quad \text{ (time-evolving PDF of the membrane potential)}, \\ F\bigl(x, t | \mathcal{H}_{t},\theta,S(t)\bigr) &\quad \text{ (time-evolving CDF of the membrane potential)}, \\ g\bigl(t | \mathcal{H}_{t},\theta,S(t)\bigr) &\quad \text{ (PDF of the spike time)}, \\ G\bigl(t | \mathcal{H}_{t},\theta,S(t)\bigr) &\quad \text{ (CDF of the spike time)}. \end{aligned}$$

All the above distributions depend on the spike history up to time *t*, denoted by $\mathcal{H}_{t}$, the parameter vector *θ* and the stimulus $S(t)$. In the following, we sometimes suppress these dependencies in the notation for readability. We write $g_{k} (t;\theta)=g(t | \mathcal{H}_{t},\theta ,S_{k}(t)) $ for the probability density of the spike time when the neuron is only presented with the single stimulus *k*.

The probability that the neuron has not yet fired at time *t*, $1-G(t)$, is equal to the probability that the membrane potential has not yet reached $x_{\mathrm{th}}$, $F(x_{\mathrm{th}}, t)$. Thus, the probability density of a spike time is given by [24, 27, 35]

8 $$ g(t) = - \frac{\partial}{\partial t} F(x_{\mathrm{th}}, t) = - \frac{\partial }{\partial t} \int_{-\infty}^{x_{\mathrm{th}}} f\bigl(x',t\bigr)\, dx' . $$

The solution of the Fokker–Planck equation provides $f(x,t)$ and $F(x,t)$, and therefore also $g(t)$. The solution of the Volterra integral equation directly provides $g(t)$ [36]. Calculating $g(t)$ enables us to do MLE, as explained in Sects. 3.5 and 3.6 below.

### Fokker–Planck Equation

The PDF of $X_{t}$ in Eq. (1) with a resetting threshold, $f(x,t)$, solves the Fokker–Planck equation, defined by the following PDE [21, 27, 33]:

9 $$ \partial_{t} f(x, t) = - \partial_{x} \bigl(b(x,t)f(x,t)\bigr) + \frac{\sigma ^{2}}{2}\partial_{xx}^{2} f(x,t), $$

with absorbing boundary condition $f(x_{\mathrm{th}}, t)=0$ and initial condition $f(x, 0)=\delta(x-x_{0})$. To solve the equation numerically we also impose a reflecting boundary condition at a small value $x=x^{-}$, where the flux equals 0: $J(x^{-}, t) = -b(x^{-},t)f(x^{-},t) + \sigma^{2} \partial_{x}f(x^{-},t) / 2=0$. We call this method the Fokker–Planck PDF method.

Another approach is to formulate the PDE for the CDF, i.e., $F(x,t)$ [27, 35] (see Appendix A.2):

10 $$ \partial_{t} F(x, t) = - b(x,t)\partial_{x}F(x,t) + \frac{\sigma ^{2}}{2}\partial_{xx}^{2} F(x,t), $$

with equivalent boundary conditions: $\partial_{x}F(x_{\mathrm{th}}, t)=0$, $F(x^{-}, t)=0$, and initial condition: $F(x, 0)=H(x-x_{0})$, where $H(\cdot)$ is the Heaviside step function. This is then called the Fokker–Planck CDF method.

Both PDEs are solved numerically using the Crank–Nicholson finite difference method, together with the Thomas algorithm efficiently solving tridiagonal systems [37]. Whichever method we use, we can always obtain the PDF (CDF) from the CDF (PDF) by numerical differentiation (integration).

### Volterra Integral Equation

The first-kind Volterra IE (Fortet equation) combines the first-passage time PDF $g(t)$ with the threshold-free membrane potential PDF $f^{*}(x, t | v, s)$ using the law of total probability [29, 30]:

11 $$ f^{*}(x_{\mathrm{th}}, t | x_{0}, 0) = \int_{0}^{t} f^{*}(x_{\mathrm{th}}, t | x_{\mathrm{th}}, s) g(s) \, ds. $$

For the OU model (1), the threshold-free PDF $f^{*}(x, t | v, s)$ is Gaussian [33, 38]:

12 $$ f^{*}(x, t | v, s) = \frac{1}{\sqrt{2\pi V(t|s)}} \exp \biggl\{ - \frac{ (x - M(t|v,s))^{2} }{2V(t|s)} \biggr\} , $$

with mean

13 $$ M(t|v,s) = v e^{-\gamma(t-s)} + \int_{s}^{t} I_{\mathrm{total}}(u) e^{-\gamma (t-u)}\, du $$

and variance

14 $$ V(t|s) = \frac{\sigma^{2}}{2 \gamma}\bigl(1 - e^{-2 \gamma(t-s)}\bigr). $$

The total current is denoted by $I_{\mathrm{total}}(t) = \gamma\mu+I(t)+H(t)$.

The initial condition for the IE is $g(0)=0$. Using this, we can solve the equation recursively and obtain $g(t)$.

The second-kind Volterra IE is defined by [39]

15 $$ g(t) = -2\psi(x_{\mathrm{th}}, t | x_{0}, 0) + 2 \int_{0}^{t} \psi(x_{\mathrm{th}}, t | x_{\mathrm{th}}, s)g(s) \, ds, $$

where

16 $$\begin{aligned} \psi(x, t | v, s) & = \partial_{t} \int_{-\infty}^{x}f^{*}\bigl(x', t | v, s \bigr) \, dx' \\ & = f^{*}(x, t | v, s) \biggl[ \gamma x - I_{\mathrm{total}}(t) - \frac{\sigma ^{2}}{2V(t|s)} \bigl(x - M(t | v, s)\bigr) \biggr]. \end{aligned}$$

A modification of $\psi(x, t | v, s)$ is proposed to avoid a singularity when $t \to s$ [36, 39] (see Appendix A.3):

17 $$ \phi(x, t | v, s) = \frac{1}{2} f^{*}(x, t | v, s) \biggl[ \gamma x - I_{\mathrm{total}}(t) - \frac{\sigma^{2}}{V(t|s)}\bigl(x - M(t | v, s)\bigr) \biggr]. $$

The second Volterra IE can also be solved numerically. It requires more computation time than the first-kind, but has higher accuracy.

### Computational Time Complexity

For both the Fokker–Planck PDE and the Volterra IE methods, the time complexity is directly related to the grid size for the numerical solution. Specifically, suppose that the grid size of the time discretization is *n* and the size of the space discretization is *m*. Then the Fokker–Planck method has complexity on the order of $O(m n)$ and the Volterra method is on the order of $O(n^{2})$ (native implementation requires $O(n^{3})$, but techniques are applied to reduce the complexity to $O(n^{2})$; see [36]). Furthermore, the computation is largely affected by the response kernel used. A discretization is applied to approximate the nonlinear kernel by a piecewise constant function with sufficiently small segmentation length. The values of the constant segments are calculated and stored in a data vector when the parameters are updated. Then inside an optimization loop, the kernel function is evaluated by referring to this data vector.

### Marginal Likelihood of the Probability-Mixing Model

Under the probability-mixing model, the marginal likelihood function of the *i*th spike train $d_{i}=(t_{1}^{i}, \ldots, t_{N_{i}}^{i})$ for a mixture of *K* stimuli is given by

18 $$ L(\theta; d_{i}) = \sum_{k=1}^{K} \alpha_{k} \prod_{j=1}^{N_{i}} g_{k} \bigl(t_{j}^{i} ; \theta\bigr), $$

and thus the marginal log-likelihood of all *N* spike trains $D=(d_{1}, \ldots, d_{N})$ is

19 $$ \ell(\theta; D) = \sum_{i=1}^{N} \log \Biggl( \sum_{k=1}^{K} \alpha_{k} \prod_{j=1}^{N_{i}} g_{k} \bigl(t_{j}^{i} ; \theta\bigr) \Biggr). $$

Marginal refers to the observed data *D*; see Sect. 3.5.1 below for a definition of the full data. MLEs are then obtained by maximizing (19). The log-likelihood function consists of logarithms of sums, and the calculations are prone to encounter numerical over- or underflow issues. To overcome this, we apply the log-sum-exp formula [37].

#### Optimizing the Likelihood Using the Expectation-Maximization Algorithm

As an alternative to optimizing directly the log-likelihood function (19), the EM algorithm [40] is well suited to solve optimization problems for mixture models and is simple to implement. The EM algorithm treats the unknown stimulus mixture component which the neuron responds to as unobserved data, or latent variables. We write $Y=(y_{1}, \ldots, y_{N})$ where $y_{i} \in\{1, 2, \ldots, K\}$, for the latent variables indicating which single stimulus each spike train is responding to. The full data then include both the observed spike trains *D* and the unobserved stimuli response *Y*.

The EM algorithm is an iterative procedure. In each iteration, the expectation of the full data log-likelihood conditional on the parameters from the previous iteration, is maximized to obtain the optimal parameters for the current iteration. The algorithm runs until convergence, i.e., the difference of parameter estimates is sufficiently small between two adjacent iterations. We use the notation *θ* for the current parameter to estimate, and $\theta_{-1}$ for the parameter estimated in the previous iteration, and likewise for the components of the probability vector *α*, i.e., $\alpha_{k}$ and $(\alpha_{k})_{-1}$.

In each iteration, the conditional expectation of the full data log-likelihood is (see Appendix A.1 for the derivation),

20 $$\begin{aligned} Q(\theta| \theta_{-1}) & = \mathbb{E} \bigl[ \log L_{c}(\theta; D, Y) | \theta_{-1}, D \bigr] \\ & = \sum_{i=1}^{N} \Biggl[ \sum _{k=1}^{K} P(y_{i}=k | \theta_{-1}, d_{i}) \Biggl( \log\alpha_{y_{i}} + \sum _{j=1}^{N_{i}}\log g\bigl(t_{j}^{i}|y_{i}, \theta\bigr) \Biggr) \Biggr], \end{aligned}$$

where the conditional probability is obtained using the Bayes formula:

21 $$ P(y_{i}=k | \theta_{-1}, d_{i}) = \frac{(\alpha_{k})_{-1} \prod_{j=1}^{N_{i}} g(t_{j}^{i}|y_{i}=k, \theta_{-1}) }{\sum_{l=1}^{K} (\alpha_{l})_{-1} \prod_{j=1}^{N_{i}} g(t_{j}^{i}|y_{i}=l, \theta_{-1}) }. $$

The EM algorithm requires the calculation of the likelihood of the spike train for all components in the mixture. Thus, the EM algorithm has (approximately) the same time complexity regarding the number of evaluations of density functions as the calculation of the marginal likelihood.

### Likelihood of the Response-Averaging Model

In the response-averaging model, the neuron responds to a weighted average of stimuli, and the model does not follow a probability mixture. The likelihood is given by

22 $$ L(\theta; D) = \prod_{i=1}^{N} \prod _{j=1}^{N_{i}} g \bigl(t_{j}^{i} ; \theta\bigr), $$

where $g(t)$ is now the probability density of spiking at time *t* when the neuron is responding to a weighted average of all *K* stimuli, $\sum_{k=1}^{K}\beta_{k}S_{k}(t)$.

### Model Checking: Uniformity Test

The goodness-of-fit can be verified by uniformity tests using the CDF $G(t)$ for all spike times in *D*. If the model perfectly describes the data, then the residuals

23 $$ z_{j}^{i} = G\bigl(t_{j}^{i}\bigr) $$

follow a standard uniform distribution, $z_{j}^{i} \sim\mathrm{U}(0,1)$. We then merge all the residuals for a specific model, and test the residuals against the uniform distribution. Quantile–quantile (QQ) plots and the Kolmogorov–Smirnov (KS) test can be employed to check for uniformity.

## Simulation Study

To illustrate the approach, we first detail the simulation study of the bursting kernel and the sinusoidal stimulus. Then results using the other types of kernels and stimuli are briefly illustrated and summarized.

Traces from model (1) using the bursting response kernel shown in Fig. 2(a), and one of the two sinusoidal stimuli shown in Fig. 2(b) or a mixture thereof was simulated according to the Euler–Maruyama scheme with a time step size of 0.1 ms. The process was run until reaching the threshold $x_{\mathrm{th}}$ where the time was recorded. The process was then reset to $x_{0}$ and started again, while the stimulus continued without any interruption, and the previously recorded spike times entered in the calculation of the post-spike currents. This was continued until the spike train was 4 s long, containing around 60 to 70 spikes. Table 1 shows the values of the parameters used for simulation and numerical computation.

**Table 1:** **Parameter values used in the simulation study**

Category	Parameter	Value	Explanation
Sinusoidal stimulus	$s_{1}$	(10,12,1,50)	First stimulus
	$s_{2}$	(20,8,0,50)	Second stimulus
Unknowns to estimate	*η*	(50,25,40,15)	Bursting response kernel
	*α*	(0.4,0.6)	Probability mixing
	*β*	(0.4,0.6)	Response averaging
	*μ*	0.5	Reversal potential
	*σ*	1	Diffusion parameter
Numerical computation	Δ*t*	0.002	Time discretization
	Δ*x*	0.02	Space discretization
	$x^{-}$	0	Lower reflecting boundary
Neuronal characteristics	$x_{0}$	0.4	Reset potential
	$x_{\mathrm{th}}$	1	Spike threshold
	*γ*	100	Decay rate

Parameter estimation was split in two, in agreement with how a typical experiment would be conducted. First we simulated spike trains responding to single stimuli. Note that in this case the probability-mixing and the response-averaging models are the same, and $\alpha=\beta=1$ are one-dimensional. The data set contains 10 spike trains, with five attending the first single stimulus and the other five attending the second single stimulus. Using this data set, we estimated parameters of the response kernel, *η*, and parameters of the diffusion model, *μ* and *σ*.

Second, we simulated spike trains using a mixture of the two sinusoidal stimuli. Two data sets were simulated, one data set consisting of 10 spike trains following the probability-mixing model, and another data set consisting of 10 spike trains following the response-averaging model. To check if the two models could be distinguished, we fitted the data using the probability-mixing model and the response-averaging model on both data sets, resulting in four combinations. During this stage, we fixed the response kernel parameters *η* to values estimated in the first step, and estimated again *μ*, *σ*, as well as *α* or *β*, depending on the model. There are therefore two sets of estimates of *μ* and *σ* for each trial. The purpose is threefold; first of all, these parameters might slightly drift in a real neuron when changing the stimulus (even if we do not change them in the simulation); second, it is of interest to understand the statistical accuracy and uncertainty of these parameter estimates when inferred in the two experimental settings; and third, comparing estimates from both single stimulus and stimulus mixtures can serve as model control, as explained below. When fitting the probability-mixing model on the data generated from this same model, we used both the marginal MLE and the EM algorithm. The above simulation and estimation procedure was repeated 100 times, generating 100 sets of estimates.

The simulation study serves different purposes. First, the four numerical methods to obtain the PDFs of the spike times, namely the first Volterra, second Volterra, Fokker–Planck PDF, and Fokker–Planck CDF, should be evaluated and compared. This is done on single stimulus spike train data. Second, the quality of the parameter estimates should be assessed, as well as how important it is to use the correct model for the estimation. This is conducted using spike trains simulated from stimulus mixtures. Also the performance of the marginal MLE and the EM algorithm in the case of the probability-mixing models should be compared. Third, it should be evaluated if it is possible to detect which of the two models generated the data. Results from these three analyses are presented in the following.

### Numerical Solutions of the Partial Differential and Integral Equations

**Fig. 3:**
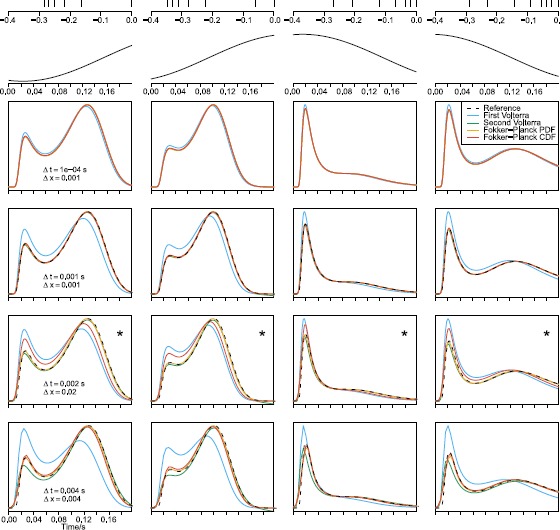
Four example ISI probability density functions, $g(t)$, calculated with four methods using different grid sizes. The *column panels* show the four different ISIs, with the spike history indicated in the *top* (with different times axes) of each column, and the sinusoidal stimulus for the corresponding time periods. The *panels in the four lower rows* show solutions of the different PDEs and IEs using increasing grid sizes in each row. In the *three lower rows*, the density function from the panels above using the second Volterra method with high accuracy is plotted as the *reference* line. As expected, the solutions become less accurate as the grid size increases. The *second row from the bottom*, *indicated with a star in the upper right corner*, shows the grid size used for estimation in the main analysis, which leads to decent approximations for all four methods

Figure 3 shows the PDFs of four example ISIs, i.e., for four different histories of past spikes, calculated by the four numerical methods, first Volterra, second Volterra, Fokker–Planck PDF and Fokker–Planck CDF, under single stimulus trials. Time has been set to 0 at the last spike time. The examples are taken from a spike train attending to the single stimulus $s_{1}$. Each column shows one example ISI, with the spike history indicated above the column (with different time axes) and the corresponding sinusoidal stimulus (same time axes as the PDFs), for four different grid sizes. The four boxed panels in each column show the solutions of the PDEs and the IEs for the ISI on top. A reference dashed black line obtained with high accuracy has been added in all panels for comparison. The grid size is given by Δ*t* for the time discretization, and Δ*x* for the space discretization, and varies from row to row. As expected, for large grid sizes (small number of bins), the performance of the four methods differ (see the three lower rows of boxed panels), but the four results converge for decreasing grid sizes (see the upper row of boxed panels). We find that the first Volterra method is more sensitive to the grid size, while the Fokker–Planck PDF method is the most robust. In the parameter estimation below, we use $\Delta t = 0.002~\mbox{s}$ and $\Delta x=0.02$ shown in the row indicated with a star.

**Fig. 4:**
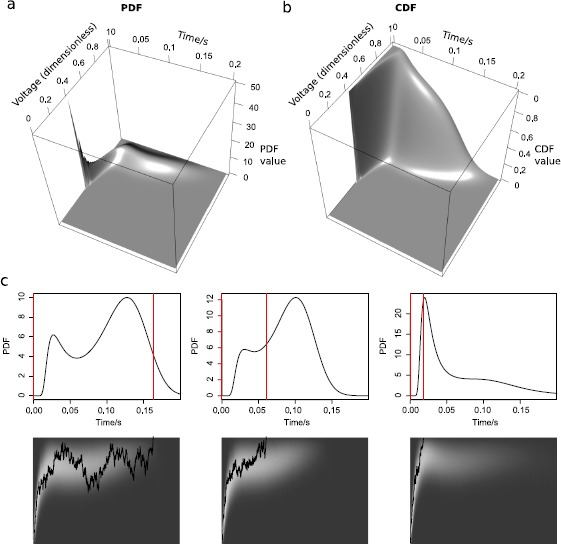
Solutions of the PDEs and the IEs and example traces. The time-evolving **(a)** PDF, $f(x,t)$, and **(b)** CDF, $F(x,t)$, from the solutions of the Fokker–Planck equation for the ISI in the left column of Fig. 3. **(c)** Three example ISIs taken from the left, middle left and middle right columns of Fig. 3. The *upper panels* show the PDFs with *red lines* indicating the spike times. The *lower panels* show the time-evolving voltage PDFs as a heat image together with the realization of the voltage path. The *brighter region* in the heat image corresponds to larger PDF values. The time when the voltage trace hits the threshold in the heat image corresponds to the spike time shown in the *upper panel* as a *red line*. Note that in the *upper panel*, the time intervals with larger ISI PDF values are where the probability (*bright region*) flows faster out of the threshold in the *lower panel*

Figure 4(a) and (b) show the time-evolving PDF and CDF of $X_{t}$ from the numerical solutions of the Fokker–Planck equation, for the ISI of the first column of Fig. 3. Time has been set to 0 at the last spike time. At 0, the PDF equals the (discretized) Dirac delta function, and the CDF equals the Heaviside step function, since at spike times, the voltage always resets to a fixed value, $x_{0}$. As time increases, the PDF shows how the probability flows out at the threshold; and the CDF at the voltage threshold illustrates the survival probability.

Figure 4(c) shows in the upper panels three examples of spike times PDFs, $g(t)$, and the lower panels show a corresponding example trace for each, plotted on top of their time-evolving PDFs of $X(t)$, $f(x,t)$, as heat-images. The three ISIs are taken from the left, middle left, and middle right panels of Fig. 3.

### Results from Single Stimulus Trials

**Fig. 5:**
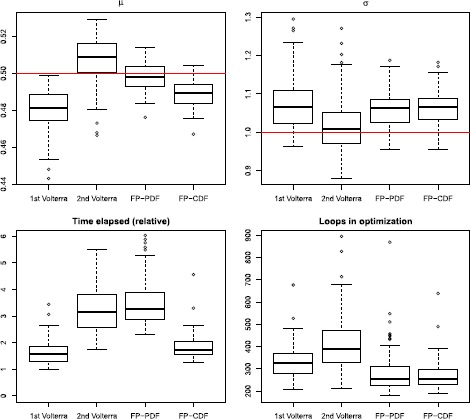
Parameter estimates and computational time. *Upper panels*: Box-plots of parameter estimates for *μ* (*left*) and *σ* (*right*) from 100 repetitions of single stimulus data. The *red lines* are the true values used in the simulations. *Lower panels*: The time elapsed and number of loops for the optimization

Parameter estimates of *μ* and *σ* from the 100 repetitions are shown in Fig. 5 as box-plots. In the lower panels, the time elapsed and the number of loops for optimization are also plotted. The means and standard deviations of parameter estimates are given in Table 2. The first Volterra method is less stable and less accurate, which is expected due to the lower accuracy in solving the spike time PDFs shown in Fig. 3. The second Volterra performs best for the estimation of *σ*, and the Fokker–Planck PDF performs best for *μ*, while the Fokker–Planck CDF does not perform as well as any of the two. On the other hand, the first Volterra and the Fokker–Planck CDF are less computational expensive. The Fokker–Planck CDF method is used in later analysis of stimulus mixtures, considering both accuracy and efficiency, though the Fokker–Planck PDF with a finer grid is used when performing KS-tests for model selection below. We also find that different methods result in different systematic estimation bias. When estimating *μ* some methods tend to overestimate and others tend to underestimate, whereas when estimating *σ* all methods have a tendency to overestimate.

**Table 2:** **Average** **±** **standard deviation of 100 parameter estimates from single stimulus data**

	*μ*	*σ*
True value	0.5	1
First Volterra	0.4800 ± 0.01095	1.076 ± 0.06913
Second Volterra	0.5066 ± 0.01287	1.020 ± 0.07281
Fokker–Planck PDF	0.4981 ± 0.00730	1.060 ± 0.04567
Fokker–Planck CDF	0.4889 ± 0.00698	1.065 ± 0.04442

**Fig. 6:**
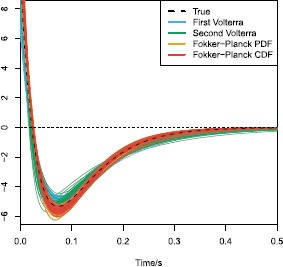
Estimates of the response kernel from 100 simulated data sets fitted to single stimulus data with the four numerical methods, each method has its own color. The *dashed black curve* is the true kernel used in the simulations

In Fig. 6, the 100 estimated response kernels from the four methods are plotted together as colored lines. The parameters of the kernel are in practice not identifiable, so we evaluate by plotting the shape of the kernel function. All methods achieved good results, capturing the overall shape. The two PDE methods obtained slightly better results, whereas the IE methods are systematically biased.

**Fig. 7:**
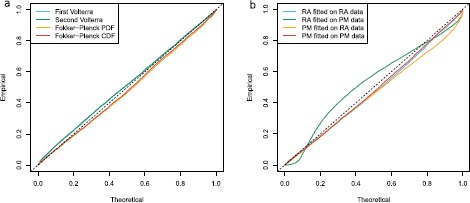
Model control. **(a)** QQ plots of the uniform residuals calculated using the transformation in Eq. (23) for the four methods fitted on single stimulus data and a grid size of $\Delta t=0.002~\mbox{s}$ and $\Delta x = 0.02$. The uniform residuals are pooled together from all 100 repetitions of the simulations. The bias is different for PDE methods and for IE methods, seen from how the points deviate from the identity line. **(b)** QQ plots of the uniform residuals of the probability-mixing (PM) model and the response-averaging (RA) model fitted on data simulated from both models responding to a stimulus mixture. For example, RA fitted on PM data means fitting the response-averaging model to data simulated from the probability-mixing model. From the QQ-plots a wrong model can be rejected

In Fig. 7(a) are QQ-plots of the uniform residuals calculated using the transformation from Eq. (23) for the four methods. The uniform residuals are pooled together from all 100 repetitions. Again, all four methods are competitive but biased, with a different bias for PDE methods and for IE methods. This bias, arising from the numerical approximations, has to be taken into account when later testing which model generated the data, forcing us to use a finer and computationally more expensive grid size.

### Distinguishing Between Response-Averaging and Probability-Mixing

**Fig. 8:**
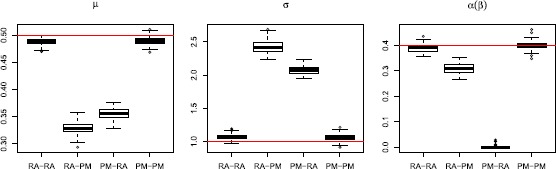
Parameter estimates of the probability-mixing (PM) model and the response-averaging (RA) model fitted to data simulated from both models responding to a stimulus mixture. For example, PM-RA means parameter estimates of the probability-mixing model fitted to data simulated from the response-averaging model

**Fig. 9:**
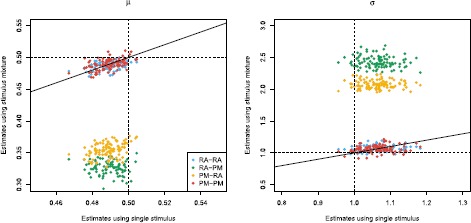
Estimates of *μ* and *σ* estimated from stimulus mixture data under either the probability-mixing or the response-averaging model plotted against the estimates from single stimulus data, for 100 repetitions. The *straight lines* are identity lines, the *dashed lines* are the true values used in the simulations. *Different colors* differentiate which model is fitted on which data for the stimulus mixture. The estimates from a stimulus mixture differ significantly from the estimates from a single stimulus when the model is wrong

The following results show that the two models can be distinguished for parameter values such that the two models are sufficiently different, which will be defined below in Sect. 4.6. Each model is fitted using the Fokker–Planck CDF method, both on data simulated according to the correct model as well as the wrong model. Figure 8 shows the estimation of *μ*, *σ*, and *α* or *β*, depending on the model, and Table 3 reports the means and standard deviations of estimates. Accurate estimation is achieved only if we apply the correct model to the corresponding data, the wrong model fitted to data generated by the other model clearly shows bad results. This implies that it is important to use the correct model for reliable inference, but we can also use this to distinguish the two models. If estimates of *μ* and *σ* change considerably from estimation on single stimulus data to estimation on stimulus mixture data, then one should suspect that the used model is wrong. This is illustrated in Fig. 9, where scatterplots of estimates from stimulus mixture data assuming a specific model is plotted against estimates from single stimulus data. The straight lines are identity lines. When the correct model is used, estimates are clustered around the identity line, but clearly separated away from the identity line if the model used for fitting is wrong. To formalize the model selection procedure, QQ plots of uniform residuals using Eq. (23) from all 100 repetitions are shown in Fig. 7(b), where points away from the identity line indicate the model is wrong. The lines for the wrong model selections are clearly worse than the correct models, but even the correct models show a significant deviation from the identity lines, which would turn out as also the correct model being rejected in a KS-test. This is most probably due to the numerical approximations, as also seen in Fig. 7(a). To check this, we conducted the same estimation procedure with the Fokker–Planck PDF method using a finer grid of $\Delta t = 0.0005~\mbox{s}$ and $\Delta x = 0.01$, and repeated for 20 times. Results are reported in Table 4, where it is clear that with a finer grid, the KS-test works as desired with high power to detect deviations from the correct model. We suggest that for parameter estimation a very fine grid is not needed, whereas for model control, the numerical approximation of the spike time PDF has to be precise. To conclude, the two models are distinguishable for the parameter settings explored here.

**Table 3:** **Average** **±** **standard deviation of 100 parameter estimates using the response-averaging (RA) model and the probability-mixing (PM) model on data sets simulated according to the two models**

	*μ*	*σ*	$\alpha_{1}$ (PM)/$\beta_{1}$ (RA)
True value	0.5	1	0.4
RA on RA data	0.4876 ± 0.00658	1.067 ± 0.04441	0.3888 ± 0.01564
PM on RA data	0.3553 ± 0.01087	2.077 ± 0.06482	0.0017 ± 0.00467
RA on PM data	0.3288 ± 0.01191	2.429 ± 0.09216	0.3098 ± 0.02161
PM on PM data (Marginal)	0.4891 ± 0.00844	1.062 ± 0.05609	0.4013 ± 0.01636
PM on PM data (EM)	0.4889 ± 0.00813	1.063 ± 0.05410	0.3988 ± 0.01012

**Table 4:** **Rejection (** $\pmb{p<0.05}$ **) rate based on the Kolmogorov–Smirnov test for uniformity done on each repetition**

Method	Low accuracy^*^	High accuracy^**^
RA on RA data	32/100	1/20
RA on PM data	100/100	20/20
PM on RA data	100/100	20/20
PM on PM data	32/100	0/20

^*^Fokker–Planck CDF method with $\Delta t=0.002~\mbox{s}$ and Δ*x* = 0.02

^**^Fokker–Planck PDF method with $\Delta t=0.0005~\mbox{s}$ and Δ*x* = 0.01

### Probability-Mixing with EM

**Fig. 10:**
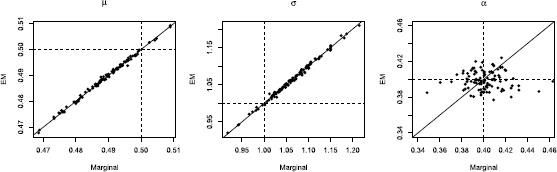
Scatter plots of the estimates using the EM algorithm against MLE with the marginal probability for 100 repetitions. The *dashed lines* are the true values used in the simulations. The two methods give almost the same results for *μ* and *σ*, whereas some zero-mean random fluctuations are seen for *α*. In this case, the EM algorithm appears to be the most precise

In the previous section, the marginal MLE was used when fitting the probability-mixing model. Here we compare the performance of the marginal MLE and the EM algorithm on the probability-mixing model fitted to the corresponding data. Figure 10 shows scatterplots of estimates obtained by the two methods, and the last two rows in Table 3 show the means and standard deviations. The two methods provide similar results, and have the same accuracy for all three parameters. However, the variance of the EM algorithm is slightly smaller, particularly for *α*. The computational burden in one loop of the numerical optimization for the two methods is approximately the same.

### Generalizations

In this section we only apply the Fokker–Planck CDF method and analyze the model for different types of response kernels and stimuli.

**Fig. 11:**
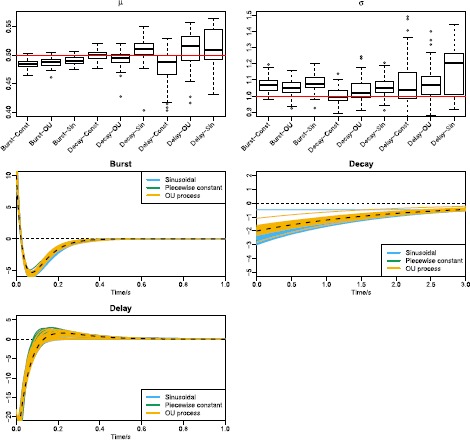
Parameter estimates of single stimuli for different combinations of response kernels and stimuli. *Top panels* show the estimates of *μ* (*left*) and *σ* (*right*) as box plots. The *x*-*axis* shows the nine combinations, for example Burst-Const means the burst kernel with a piecewise constant stimulus, Delay-OU means the delay kernel with a stochastic stimulus generated by the OU process, and so on. The delay kernel induces the largest variance in parameter estimates. *Middle* and *bottom panels* show the estimates of the three types of response kernels. *Different colors* distinguish between the three stimulus types

*Single stimulus*. We analyze nine combinations of response kernels and stimuli. For each combination we simulate 10 spike trains following one single stimulus. Figure 1 shows the combinations and the realizations of spike trains. On these spike trains parameters and response kernels are estimated. The simulations are then repeated 100 times. For the stochastic stimulus, we use a single realization so that the stimulus is identical in all repetitions and the statistical performance of the estimators can be assessed. The estimates of parameters and response kernels are shown in Fig. 11. The estimates using the delay kernel have larger variance, possibly due to our specific choice of kernel parameters that makes the spiking rate less sensitive to stimulus strength (see bottom panels of Fig. 1). The estimates of parameters and kernels for all combinations are acceptable. The parameters used for the response kernels and stimuli are shown in Table 5.

**Table 5:** **Parameter values for all response kernels and stimuli used in the single stimulus study for the generalized analysis**

	Category	Parameter value
Stimulus, *s*	Sinusoidal	(10,12,1,50)
	Piecewise constant	(50,70,50,30,50,60,0,1.3,1.7,2.3,2.7,3.8,5)
	OU process	(50,20)
Response kernel, *η*	Bursting	(50,25,40,15)
	Decay	(0,0,2,0.5)
	Delay	(20,8,50,15)

**Fig. 12:**
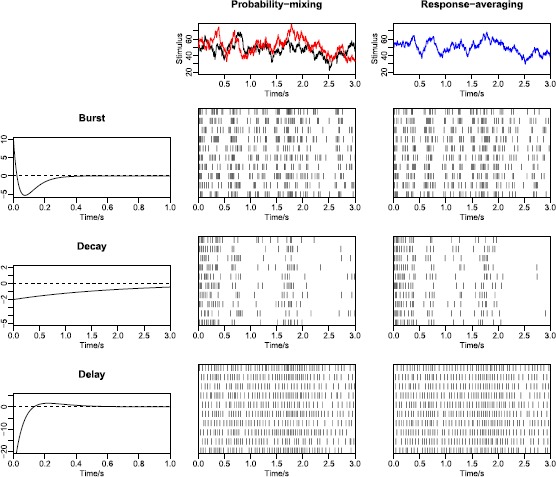
Realization of spike trains for a stimulus mixture consisting of two OU processes for three types of response kernels, assuming either probability-mixing (*left*) or response-averaging (*right*). In the *top panels*, the *left* shows the two stimuli, and the *right* shows the weighted average of the two. For the 10 spike trains simulated from the probability-mixing model, four respond to the same stimulus and six respond to the other

**Fig. 13:**
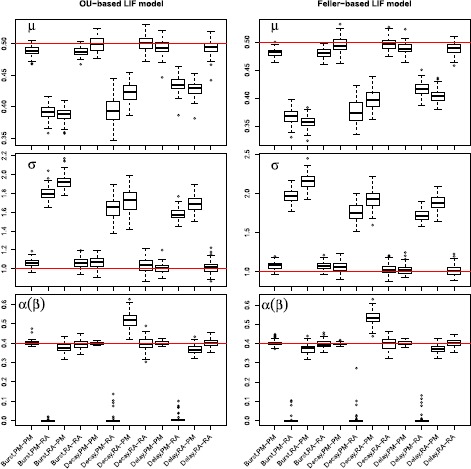
Parameter estimates for a stimulus mixture consisting of two OU processes for three types of response kernels, assuming either probability mixing or response averaging. In the *left panel* is shown the estimates of the OU-based LIF model, and in the *right panel* is shown the Feller-based LIF model. In both *left and right panels*, the *x*-*axis* shows 12 cases combining response kernels, probability mixing and response averaging. For example, Decay, PM-RA means fitting the probability-mixing model to data simulated from the response-averaging model, using the decay kernel

*Stimulus mixtures*. We use two OU processes as stimuli, and apply all three types of response kernels. The top panels of Fig. 12 show the two stochastic stimuli, and their weighted average. The latter is what neurons respond to according to the response-averaging model. For each combination, we simulate 10 spike trains, using identical stimuli in each repetition. Results are shown in the left panels of Fig. 13, where both the probability-mixing (PM) model and the response-averaging (RA) model are fitted to data generated from both models. When fitting the probability-mixing model, only the EM algorithm is applied. We employ the same strategy as in the main analysis: we first estimate parameters on data generated from single stochastic stimuli, and then fix the response kernel and estimate the other parameters on data generated from stochastic stimulus mixture. The results for all three kernels on a stochastic stimulus mixture are the same as the main analysis above using the bursting kernel and sinusoidal stimuli: we obtain accurate estimates of all parameters only if we apply the correct model to the corresponding data.

*State dependent noise*. Finally, the diffusion term in the LIF model (1) was modified to include the square root of $X(t)$ as in the Feller model [41–43], yielding

24 $$ X(t) = \bigl(-\gamma\bigl(X(t) - \mu\bigr) + I(t) + H(t) \bigr)\, dt + \sigma \sqrt{X(t)}\, dW(t). $$

The same analysis as in the previous section was repeated using two OU processes as stimuli and three types of response kernels. Results are shown in the right panels of Fig. 13, which are almost the same as the results using the original LIF model shown in the left panels.

**Fig. 14:**
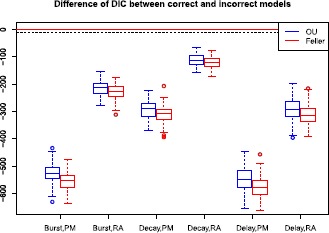
Difference of DIC between correct and incorrect models. We calculate the difference of DIC between fitting the correct model to the corresponding data and fitting the incorrect model to the same data, and plot the difference as box-plots for 100 repetitions. The *x*-*axis* shows different combinations of kernel and data. For example, Burst, PM means the difference of DIC between using correct model (PM) and incorrect model (RA) on PM data, under the burst kernel. Likewise, Delay, RA means the difference of DIC between using correct model (RA) and incorrect model (PM) on RA data, under the delay kernel. *Blue* stands for the OU-based LIF model and *red* stands for the Feller-based model. A difference of −10 is shown as a *dashed line*. A difference greater than ±10 is regarded as strong evidence of supporting one model over the other [44]

*Model selection*. In stimulus mixture analysis, model selection is conducted for both the OU-based and the Feller-based LIF models. In Fig. 14 we compare the deviance information criterion (DIC) between the correct and the incorrect model. The DIC difference equals −2 times the difference of the log-likelihoods, because the two models have the same number of parameters. The correct model is strongly supported in every case. Table 6 shows rejection ($p<0.05$) ratios using KS-tests for all combinations in the stimulus mixture analysis. We also tried other pairs of stochastic stimulus mixtures (results not shown) and found that the more similar the two stimuli are, the more the rejection ratios tend to decrease, whether using the correct or the incorrect model, and if two stimuli are more different, all rejection ratios tend to increase, including rejections of the true model. Finally, as expected the KS-test rejection ratio is sensitive to data size: using smaller number of spike trains reduces the rejection ratio. In particular, the rejection of fitting the PM model to RA data (PM-RA) with the decay kernel, and fitting the RA model to PM data (RA-PM) with the delay kernel, is extremely sensitive to similarity of stimuli and data size. This makes the KS-tests less robust. Thus, we recommend using the KS-tests together with other model selection methods for more reliable conclusions.

**Table 6:** **Rejection (** $\pmb{p<0.05}$ **) rate based on the Kolmogorov–Smirnov test for uniformity, using different response kernels with the mixture of stochastic stimuli**

		RA-RA	RA-PM	PM-RA	PM-PM
OU	Burst	22/100	99/100	100/100	19/100
	Decay	1/100	100/100	83/100	1/100
	Delay	30/100	77/100	97/100	34/100
Feller	Burst	23/100	100/100	95/100	22/100
	Decay	0/100	100/100	81/100	1/100
	Delay	30/100	84/100	100/100	37/100

Results of both the OU-based and the Feller-based LIF models are shown

### Model Selection Accuracy

The results above show that parameters can be inferred and the correct model can be determined for the specific parameter choices used in the simulations. Here we explore the model selection accuracy for varying parameter values including the weight, stimulus dissimilarity, stimulus strength and number of spike trains. In the following analysis, we use the bursting response kernel, a mixture of two stochastic stimuli and the Fokker–Planck CDF method. To introduce a stimulus dissimilarity, a sinusoidal perturbation is added to one of two identical OU processes, $\tilde{S}(t) = S(t) + a\sin(10t)$, where *t* is measured in seconds and *a* is the perturbation size. To change the stimulus strength, the OU processes are linearly scaled using $\tilde{S}(t) = bS(t)$ where *b* denotes the scaling size.

**Fig. 15:**
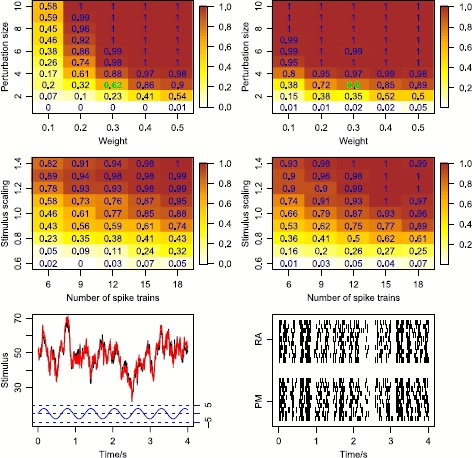
Model selection accuracy. Successful selection is defined as a DIC difference greater than 2, and the proportion of correctly identified models is calculated over 100 repetitions. Note that a not correctly identified model in most cases means that the DIC difference was smaller than 2, not that the wrong model was selected. *Top left*: proportion of correctly identified models with weights from 0.1 to 0.5 and perturbation size from 1 to 10 for RA data, using 10 spike trains. *Top right*: the same for PM data. *Middle left*: proportion of correctly identified models for number of spike trains of 6 to 18 and stimulus scaling from 0.6 to 1.4 for RA data, using a weight of 0.3 and a perturbation size of 3, shown in *green* in the *top panels*. *Middle right*: the same for PM data. *Bottom left*: the two stimuli curves (*black* and *red*) with perturbation size 3 (sinusoidal curve shown in *blue*) used for the cases shown in *green* in *top panels*. *Bottom right*: example spike trains following either RA or PM, using the two stimuli shown in the *left*, with weight 0.3

We focus on model selection accuracy without reporting parameter estimates. Model selection is denoted successful if the DIC for the true model is more than 2 smaller than the wrong model. This is the value suggested in [44] to indicate substantial empirical support for the selected model compared to the other model. Figure 15 explores model selection results as a function of parameter values, and provides an overall picture how these parameters affect model selection. The conveyed message verifies our intuition: model selection is more reliable if the stimuli are more different, the weights are more even, the stimulus difference is stronger or the sample size is larger (a larger number of spike trains). The first three make the responses of the two models more different, and the last provides more statistical power. Furthermore, the thresholds of these parameter values in terms of successful model selection are surprisingly low. A weight value of 0.2 and a perturbation size around 6 (i.e., around 10 % of the stimulus strength) are sufficient to ensure a decent selection. For a more even weight of 0.4, only a perturbation size of 3 (around 5 %) is necessary to provide good model selection for both RA and PM data. Indeed, 5 % perturbation in a stimulus is undetectable by a simple graphical inspection of the spike trains (bottom panels in the figure), but the finer statistical analysis can detect the difference between the models. Even with small weight and stimulus dissimilarity, model selection can be improved by using stronger stimuli or enlarging the sample size with more spike trains. Note that these analyses are easily generalized for a given problem at hand by first estimating the response kernel of a given neuron under a given stimulus, and then simulating data with this response kernel and stimulus, varying parameters of the two models. That will indicate for which parameter values the model selection can be trusted.

## Discussion

### Estimation of the Decay Rate

We have shown that parameter inference can be successfully conducted for the probability-mixing and the response-averaging model on corresponding data incorporating different response kernels for LIF neurons. The decay rate *γ* has been assumed known. We also attempted to estimate all parameters including *γ* (results not shown), but the optimization often finds local minima and leads to low accuracy. The estimation of *γ* seems to suffer from identifiability problems, due to only observing spike times and not the underlying membrane potential. Nevertheless, to estimate *γ* we may fix it at different values and run the optimization procedure for the rest of the parameters, and then compare the model fit for the different *γ* values. This is not pursued here.

### Bias of the Numerical Methods

We found that the parameter estimates and the QQ plots from the four methods suffer from over- and underestimation issues. The MLE is based on the first-passage time probabilities, which we obtain using four numerical methods, Fokker–Planck PDF, Fokker–Planck CDF, first Volterra and second Volterra. Because of the intrinsic differences between these methods, discretization leads to different biases of the calculated spike time PDFs. As seen from Fig. 3, when increasing the grid size, the first Volterra and the Fokker–Planck CDF methods tend to increase the PDF value in the beginning of the ISI, while the second Volterra tends to slightly decrease it. The low accuracy of the first Volterra method arises from a singularity of $f^{*}(x,t|v,s)$ when $v=x$ and $t\to s$. However, by removing the singularity the second Volterra is more accurate for numerical computations.

### Efficiency of Numerical Methods

We choose the Fokker–Planck CDF method for estimation of mixtures, because it achieves a well-behaved balance between accuracy and computational burden. Table 2 also shows that this method has the smallest variance on parameter estimates.

Although the first Volterra method is the computationally fastest, it has poor convergence, as seen from the number of loops in the bottom right panel in Fig. 5. Overall, the PDE methods tend to converge faster than the IE methods.

The performance is affected by the grid size. The estimates in Fig. 5 uses $\Delta t=0.002~\mbox{s}$ and $\Delta x = 0.02$. This discretization setting generally achieves acceptable computation times and statistical accuracy, but as shown in Sect. 4.3, a finer grid is needed for model selection. One may tweak the grid sizes in order to obtain separate settings for each of the four methods to obtain comparable efficiency and accuracy. However, considering that in practical data the errors come from many sources like measurement errors and approximate modeling, the optimal discretization on simulated data is of less importance and interest. Thus, we suggest the current setting as providing a generally good balance, and we will not investigate this further.

### EM for Better Estimation of Mixture Probabilities

Figure 10 shows that the estimation of the mixture probability parameter *α* is slightly less stable for the marginal MLE than for the EM algorithm. The EM algorithm implicitly enlarges the data size by using latent variables for the mixture probability, referred to as *data augmentation* [45]. The complete-data log-likelihood function used in the M step does not contain logarithms of sums, making the estimation more stable. By iteratively updating the expectation in the E step and obtaining stable estimation in the M step, the EM algorithm improves the stability when inferring the probability-mixing model, and in general, mixture models.

Although the EM algorithm performs better, it is only slightly better for *α* and the improvement is negligible or non-existent for *μ* and *σ*. This is because we only use two components in the mixture, which does not generate notable differences between the marginal MLE and the EM algorithm. A larger advantage of the EM algorithm can be expected under more complex stimulus mixtures. Furthermore, the response kernel is fixed, and the two methods use the same initial values for *μ* and *σ* (obtained from the single stimulus trials) in the optimization procedure, which also contributes to the similarity of results between the two methods.

### Extension of Noise

In this paper a one-dimensional stochastic differential equation model driven by a Wiener process for the membrane potential has been considered, which arises as an approximation to Stein’s model [46], leading to the OU model, or to the extended model including reversal potentials, proposed by Tuckwell [41], leading to the Feller model [42]. The model does not take into account specific dynamics of synaptic input or ion channels, which affects the dynamics, see, e.g., [47–49], where the autocorrelations of the synaptic input is shown to be an important factor. This is partially accounted for in our model through the memory kernels. Incorporating autocorrelated synaptic input or ion channel dynamics would lead to a multi-dimensional model. In principle, the first-passage time probabilities could then be obtained by solving multi-dimensional Fokker–Planck equations [24]. However, the statistical problem is further complicated by the incomplete observations, since typically only the membrane potential is measured, as studied in [50]. In even more realistic models non-Gaussian noise can be included, for example combining the diffusion process with discrete stochastic synaptic stimulus arrivals, leading to a jump-diffusion process, whose Fokker–Planck equation is generalized as an integro-differential equation [51]. Solving multi-dimensional or generalized Fokker–Planck equations are significantly more expensive and exact MLE becomes less appealing. This is not pursued here.

### The Response-Averaging Model

The response-averaging model used here is slightly different from the response-averaging model by Reynolds et al. [8]. In our model the average is calculated over the currents for each stimulus, while in their model the average is calculated over the firing rates for each stimulus. The reason is as follows. In a spiking neuron model like the LIF model, the generation of each single spike rather than the firing rate is modeled. Whether in the probability-mixing model, the response-averaging model or any other model, the spiking is affected by stimuli only through currents. Our model is formulated based on this idea, using a unified spike-generating mechanism for both the probability-mixing and the response-averaging model. The resulting firing rate averaged over a time window from a weighted average of single stimuli, will also be a weighted average of firing rates from single stimuli but with different weights. Our response-averaging model therefore provides the same consequence in terms of firing rates as the model by Reynolds et al.

### Model Selection of Probability-Mixing and Response-Averaging

We finish by addressing the possible model selection methods for probability mixing and response averaging on real data. We have shown that the probability-mixing and the response-averaging models can be clearly distinguished if fitted on simulated data. However, real data will likely not follow exactly one of the two models, but one of the models might give a better description of the data than the other. We might need to design more sophisticated methods for model checking and model selection. Apart from conducting uniformity tests based on the uniform residuals from the transformation (23), such as the KS-test as we have done, we can compare the Akaike information criterion (AIC) and Bayesian information criterion (BIC) between the two models. We have used a unified DIC method due to equal number of parameters, but AIC and BIC should be used if two models have differing numbers of parameters. Furthermore, the model can also be checked by evaluating the performance of prediction (of spikes) and decoding (of stimuli), using methods such as root mean squared deviation (RMSD) between empirical and predicted values. See [19] for the use of these approaches to distinguish between the two models on experimental data from the middle temporal visual area of rhesus monkeys.

## Appendix

### A.1 The EM Algorithm for Stimulus Mixtures

The complete likelihood for the full data $(D, Y)$ is

25 $$\begin{aligned} L_{c}(\theta; D, Y) & = \prod _{i=1}^{N} \prod_{j=1}^{N_{i}} g\bigl(t_{j}^{i},y_{i}|\theta\bigr) \\ & = \prod_{i=1}^{N} P(y_{i}| \theta) \prod_{j=1}^{N_{i}} g \bigl(t_{j}^{i}|y_{i},\theta\bigr) \\ & = \prod_{i=1}^{N} \alpha_{y_{i}} \prod_{j=1}^{N_{i}} g\bigl(t_{j}^{i}|y_{i}, \theta\bigr) . \end{aligned}$$

#### A.1.1 Expectation Step

The expectation of the full data log-likelihood conditional on the previous parameters $\theta_{-1}$ and the observed data *D* is

26 $$\begin{aligned} Q(\theta| \theta_{-1}) & = \mathbb{E} \bigl[ \log L_{c}(\theta; D, Y) | \theta_{-1}, D \bigr] \\ & = \mathbb{E} \Biggl[ \sum_{i=1}^{N} \Biggl( \log\alpha_{y_{i}} + \sum_{j=1}^{N_{i}} \log g\bigl(t_{j}^{i}|y_{i},\theta\bigr) \Biggr) \Big| \theta_{-1}, D \Biggr] \\ & = \sum_{i=1}^{N} \Biggl[ \mathbb{E} \Biggl( \log\alpha_{y_{i}} + \sum_{j=1}^{N_{i}} \log g\bigl(t_{j}^{i}|y_{i},\theta\bigr) \Big| \theta_{-1}, D \Biggr) \Biggr] \\ & = \sum_{i=1}^{N} \Biggl[ \sum _{k=1}^{K} P(y_{i}=k | \theta_{-1}, d_{i}) \Biggl( \log\alpha_{y_{i}} + \sum _{j=1}^{N_{i}}\log g\bigl(t_{j}^{i}|y_{i}, \theta\bigr) \Biggr) \Biggr]. \end{aligned}$$

The conditional probability of the latent variable is obtained from Bayes formula:

27 $$\begin{aligned} P(y_{i}=k | \theta_{-1}, d_{i}) & = \frac{ P(y_{i}=k | \theta_{-1}) \prod_{j=1}^{N_{i}} g(t_{j}^{i}|y_{i}=k, \theta_{-1})}{\sum_{l=1}^{K} P(y_{i}=l | \theta_{-1}) \prod_{j=1}^{N_{i}} g(t_{j}^{i}|y_{i}=l, \theta_{-1}) } \\ & = \frac{(\alpha_{k})_{-1} \prod_{j=1}^{N_{i}} g(t_{j}^{i}|y_{i}=k, \theta_{-1}) }{\sum_{l=1}^{K} (\alpha_{l})_{-1} \prod_{j=1}^{N_{i}} g(t_{j}^{i}|y_{i}=l, \theta_{-1}) }. \end{aligned}$$

#### A.1.2 Maximization Step

In the Maximization step, the new parameter *θ* is obtained by optimizing the conditional expectation $Q(\theta| \theta_{-1})$. A new iteration is then initiated using *θ* as the previous parameter. The loops run until *θ* and $\theta_{-1}$ are sufficiently close.

### A.2 The Fokker–Planck CDF Method

Plugging $f(x,t) = \partial_{x}F(x,t)$ into the Fokker–Planck PDE

28 $$ \partial_{t}f(x,t) = - \partial_{x} \bigl(b(x,t)f(x,t)\bigr) + \frac{\sigma ^{2}}{2}\partial_{xx}^{2}f(x,t) $$

gives

29 $$ \partial_{t}\partial_{x}F(x,t) = - \partial_{x} \biggl[ b(x,t)\partial _{x}F(x,t) - \frac{\sigma^{2}}{2} \partial_{x} \partial_{x}F(x,t) \biggr]. $$

Integrating both sides w.r.t. *x* yields

30 $$ \partial_{t}F(x,t) = -b(x,t)\partial_{x}F(x,t) + \frac{\sigma ^{2}}{2}\partial_{xx}^{2}F(x,t) + C(t). $$

Recall the lower reflecting boundary at $x=x^{-}$, where $F(x^{-}, t) = 0$ and thus $\partial_{t}F(x,t) | _{x=x^{-}} = 0$. We also see that the flux equals 0, so

31 $$\begin{aligned} J\bigl(x^{-},t\bigr) & = -b\bigl(x^{-},t \bigr)f\bigl(x^{-},t\bigr) + \frac{\sigma^{2}}{2}\partial _{x}f(x,t)|_{x=x^{-}} \\ & = -b(x,t) \partial_{x} F(x,t) | _{x=x^{-}} + \frac{\sigma^{2}}{2} \partial_{xx}^{2}F(x,t) | _{x=x^{-}} \\ & = 0. \end{aligned}$$

Thus, $C(t)=0$, and we obtain the PDE for $F(x,t)$:

32 $$ \partial_{t} F(x, t) = - b(x,t)\partial_{x}F(x,t) + \frac{\sigma ^{2}}{2}\partial_{xx}^{2} F(x,t). $$

### A.3 Removing the Singularity in the Second-Kind Volterra Equation

The singularity arises because $f^{*}(x,t | v, s)$ diverges when $v=x$ and $t\to s$. This can be resolved by the method proposed by [39]. Note that the substitution of $\psi(x, t | v, s)$ in Eq. (15) with any function of the form

33 $$ \phi(x, t| v, s) = \psi(x, t | v, s) + \lambda(t) f^{*}(x,t | v, s) $$

will also satisfy the second Volterra equation, since

34 $$\begin{aligned} p(t) =& -2 \psi(x,t|v,s) - 2 \lambda(t) f^{*}(x,t | v,s) + 2 \int _{0}^{t} \psi(x_{\mathrm{th}}, t | x_{\mathrm{th}}, s)p(s)\, ds \\ &{} + 2\lambda(t) \int_{0}^{t} f^{*}(x_{\mathrm{th}}, t | x_{\mathrm{th}}, s) p(s) \, ds \\ =& -2 \psi(x,t|v,s) + 2 \int_{0}^{t} \psi(x_{\mathrm{th}}, t | x_{\mathrm{th}}, s)p(s)\, ds, \end{aligned}$$

where we have applied the first Volterra equation, Eq. (11).

We then set $\phi(x, t |v, s)$ to 0 as $t \to s$ by letting

35 $$\begin{aligned} \lambda(t) & = -\lim_{t\to s} \frac{\psi(x,t|v,s)}{f^{*}(x,t|v,s)} \\ & = - \lim_{t\to s} \biggl[ \gamma x - I_{\mathrm{total}}(t) - \frac{\sigma ^{2}}{2V(t|s)}\bigl(x - M(t|x,s)\bigr) \biggr] \\ & = - \gamma x + I_{\mathrm{total}}(t) + \lim_{t\to s} \biggl[ \gamma\frac{x - x e^{-\gamma(t-s)} - \int_{s}^{t}I_{\mathrm{total}}(u) e^{-\gamma(t-u)}du }{1-e^{-2\gamma(t-s)}} \biggr] \\ & = -\gamma x + I_{\mathrm{total}}(t) + \gamma\lim_{t \to s} \biggl[ \frac{ gxe^{-\gamma(t-s)} - I_{\mathrm{total}}(t)e^{-\gamma(t-t)} }{2\gamma e^{-2\gamma (t-s)}} \biggr] \\ & = \frac{I_{\mathrm{total}}(t)-\gamma x}{2}. \end{aligned}$$

Then we have

36 $$ \phi(x, t | v, s) = \frac{1}{2} f^{*}(x, t | v, s) \biggl[ \gamma x - I_{\mathrm{total}}(t) - \frac{\sigma^{2}}{V(t|s)}\bigl(x - M(t | v, s)\bigr) \biggr], $$

and the singularity will be removed when $v=x$ and $t\to s$.

## Abbreviations

LIF: Leaky integrate-and-fire
PDE: Partial differential equation
IE: Integral equation
EM: Expectation-maximization
ISI: Interspike interval
MLE: Maximum likelihood estimation/estimator
PDF: Probability density function
CDF: Cumulative distribution function
QQ: Quantile–quantile
KS: Kolmogorov–Smirnov
DIC: Deviance information criterion
AIC: Akaike information criterion
BIC: Bayesian information criterion
